# Managers’ prevention and self-confidence in supporting employees with common mental disorders

**DOI:** 10.1093/eurpub/ckac131.261

**Published:** 2022-10-25

**Authors:** J Hultqvist, P Zhang, C Staland-Nyman, M Bertilsson

**Affiliations:** 1 Department of Health and Rehabilitation, University of Gothenburg, Göteborg, Sweden; 2 School of Public Health and Community Medicine, University of Gothenburg, Göteborg, Sweden; 3 School of Health and Welfare, Halmstad University, Halmstad, Sweden

## Abstract

**Background:**

Despite managers’ responsibility for work environment and employee health few studies have investigated managers’ actions to prevent common mental disorders (CMD). Concerning prevention of CMD, qualitative studies report managers feeling unconfident. We investigated managers’ self-confidence in supporting employees with CMD and two managerial preventive actions (MPA): ‘reviewing assignments and the work situation’ (MPA-review) and ‘taking initiative to talk about depression and anxiety at the workplace’ (MPA-talk). We hypothesized that managers’ self-confidence in supporting employees with CMD would be positively associated with both MPAs.

**Methods:**

An on-line survey was sent in 2017 to 4737 managers, answer rate 71% (n = 3358), of which 2 899 were included in this study. Both independent and dependent variables were measured through single questions. Self-confidence in supporting employees with CMD was analyzed in relation to MPA-review and MPA-talk using binary logistic regression analysis adjusted for sex, education, managerial experience and training, lived experiences of CMD, work organizational context and general preventive actions in the organization towards CMD.

**Results:**

The proportion of managers with higher self-confidence in supporting employees with CMD was 48.9%, performing MPA-review and MPA-talk was 50% and 57% respectively. Adjusted for all co-variates, managers with higher self-confidence in supporting employees with CMDs were more likely to do both MPA-review (OR 1.57; 95% CI, 1.31-1.87) and MPA-talk (OR 2.06; 95% CI, 1.71-2.25).

**Conclusions:**

The study hypotheses were confirmed. Managers with more self-confidence in supporting employees with CMD were more likely to take actions to prevent CMD, particularly regarding initiating talks about CMD with subordinates. The study suggests it is important to strengthen managers self-confidence in supporting employees with CMD to increase their preventive actions towards CMD.

**Key messages:**

• Managers’ self-confidence in supporting employees with CMD is vital for workplace prevention.

• To strengthen managers’ self-confidence in supporting employees with CMD is essential.

